# Severe hydronephrosis secondary to uterine artery pseudoaneurysm in the early second trimester of pregnancy: A case report

**DOI:** 10.1186/s12884-017-1529-8

**Published:** 2017-09-25

**Authors:** Tsukuru Amano, Shinsuke Tokoro, Shunichiro Tsuji, Takashi Inoue, Fuminori Kimura, Takashi Murakami

**Affiliations:** 1 Department of Obstetrics and Gynecology, Shiga University of Medical Science, Seta, Otsu, 520-2192 Japan; 2 Department of Obstetrics and Gynecology, Omihachiman Community Medical Center, Tsuchidacho, Omihachiman, Shiga 523-0082 Japan; 3 Department of Obstetrics and Gynecology, National Hospital Organization Higashi-Ohmi General Medical Center, Gokashoucho, Higashiohmi, Shiga 527-8505 Japan

**Keywords:** Uterine artery pseudoaneurysm, Pregnancy, Hydronephrosis, Internal iliac artery ligation, Case report

## Abstract

**Background:**

Uterine artery pseudoaneurysm (UAP) normally presents genital bleeding in the puerperal period, and severe hydronephrosis rarely presents during pregnancy. We report a rare case of severe ureteral obstruction accompanied by uterine artery pseudoaneurysm in the early second trimester of pregnancy, which was successfully treated by surgical intervention.

**Case presentation:**

A 42-year-old nulligravid woman who had undergone myomectomy 3 years earlier was referred to our hospital for acute left abdominal pain at the 17th week of gestation. Ultrasonography showed severe left hydronephrosis and a 6-cm mass in the parauterine space. Color Doppler ultrasonography revealed a spinning turbulent flow pattern inside the mass lesion. Contrast-enhanced computed tomography revealed the left uterine artery feeding blood flow to the mass and left ureteral obstruction by the mass. These results indicated left hydronephrosis secondary to left uterine artery pseudoaneurysm. To resolve the problem, laparotomy was performed. As uterine artery isolation was impossible, ligation of the left internal iliac artery and releasing of the ureteral obstruction were carried out. The hydronephrosis and abdominal pain promptly resolved after the surgery. Thereafter, fetal development proceeded normally in the remaining months of the pregnancy. A healthy baby was delivered through cesarean section at 36 weeks gestational age. At the cesarean section, the left lower uterine segment where the UAP had been present was not visible because of the firm adhesion in around it.

**Conclusions:**

Uterine artery pseudoaneurysm can cause hydronephrosis in the early second trimester of pregnancy. Ligation of the unilateral internal iliac artery is a safe and effective intervention to block the blood flow to the uterine artery pseudoaneurysm during pregnancy, when uterine artery ligation seems not possible. In the pregnancy after previous surgical procedures to the uterus, uterine artery pseudoaneurysm should be considered in the differential diagnosis of symptomatic hydronephrosis.

## Background

A pseudoaneurysm is defined as an extra-luminal collection of arterial blood flow that communicates with parent vessels through a defect in the arterial wall [1]. Uterine artery pseudoaneurysm is a rare complication, occurring after cesarean section, traumatic abortion, or myomectomy. UAP after myomectomy has an incidence of 1% [2]. Uterine artery pseudoaneurysm (UAP) commonly presents with genital bleeding in the puerperal period but rarely presents abdominal pain during pregnancy [3]. On the other hand, it is well known that hydronephrosis occurs widely in the second and third trimesters of pregnancy [4, 5]. However, symptomatic severe hydronephrosis secondary to UAP in pregnancy is uncommon, and uterine artery embolization or early termination are often attempted to avoid the catastrophic effects of rupture on both the mother and the fetus [6–8]. However, the optimal treatment remains unknown because of the rarity of this condition.

This is a case report of uterine artery pseudoaneurysm, which presented with abdominal pain due to severe hydronephrosis in the early second trimester of pregnancy and was successfully treated by surgical intervention.

## Case presentation

A 42-year-old nulligravid woman at 17 weeks of gestation was transferred to our hospital for strong left lower abdominal pain. She had undergone laparotomic myomectomy for multiple myomas when she was 39 years old. The total number of excised myomas was 34, and blood loss was 9024 mL. The postoperative course was uneventful, and she was discharged about a week after the operation. At the same period, she visited a cardiology clinic for mild hypertension; as a result, medication was not necessary with the diagnosis of white coat hypertension. Magnetic resonance imaging of the pelvic area 6 months after the surgery revealed no abnormality in the uterus, uterine artery, or ureters.

She subsequently became pregnant through *in-vitro* fertilization and the embryo transfer technique. Thereafter, she underwent prenatal checkups, and the pregnancy followed a normal course. At the 17th week of gestation, she felt continuous pain around the left lower abdomen, for which she was referred to our hospital.

**Fig. 1:**
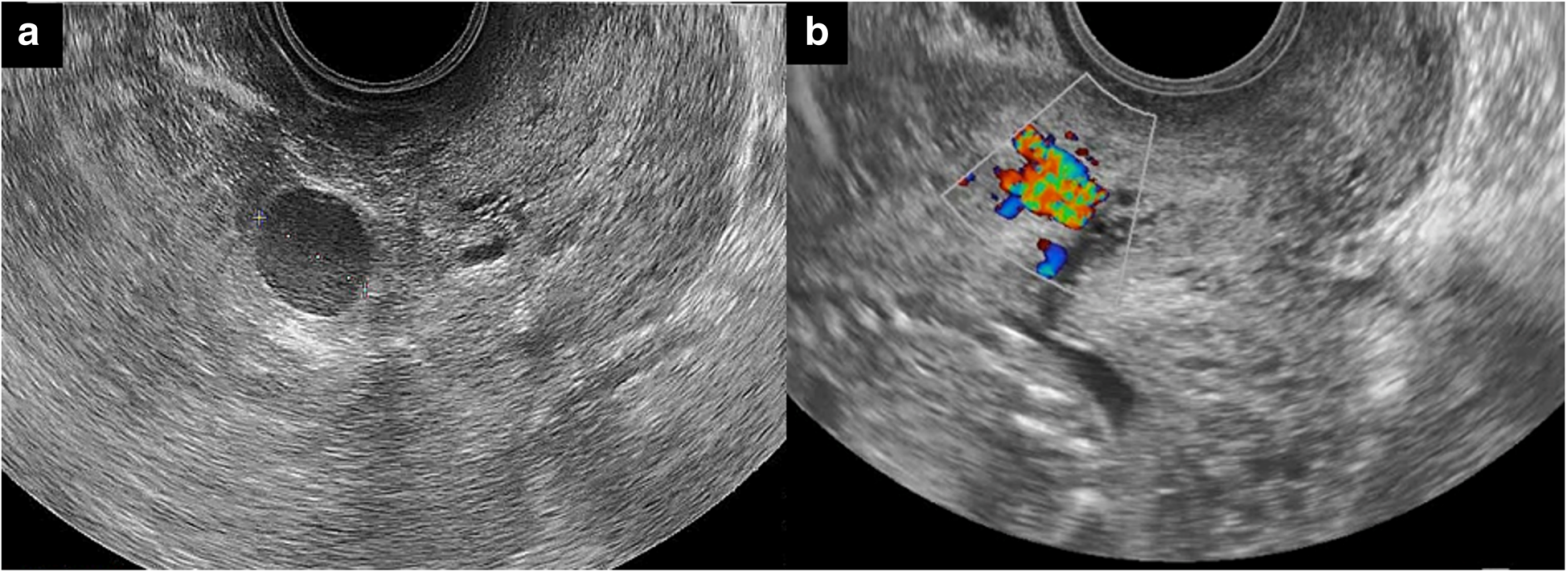
**a** Hypo-echoic coin lesion measuring 17.5 mm in diameter surrounded by hetero-echoic mass. Transvaginal ultrasonography. **b** Spinning turbulent flow pattern inside the coin lesion. Transvaginal color Doppler ultrasonography

**Fig. 2:**
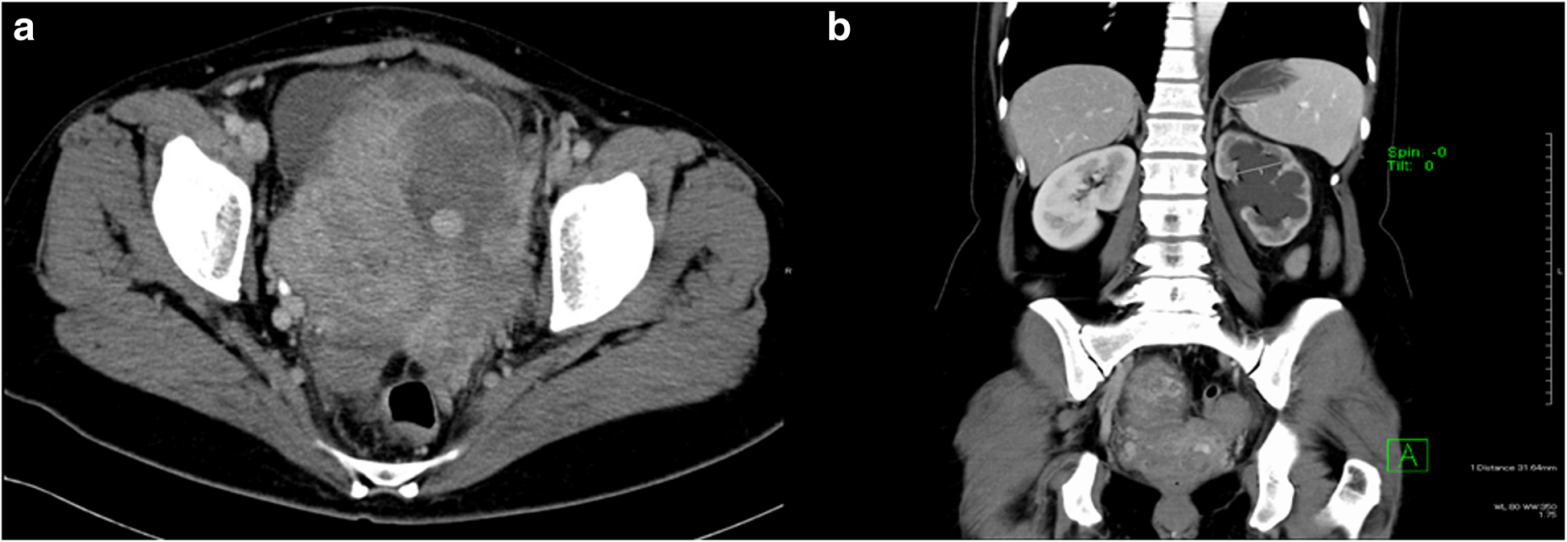
Computed tomography scan demonstrating (**a**) uterine artery pseudoaneurysm and (**b**) severe left hydronephrosis (calyceal diameter was over 31 mm)

She presented no vaginal bleeding. The laboratory data showed slight anemia and an increase of leukocytes: hemoglobin 9.8 g/dL, white blood cell count 18.1 × 10^9^/L, and platelet count 283 × 10^9^/L. The renal function was normal: serum creatinine 0.57 mg/dL, blood urea nitrogen 9.7 mg/dL. Fetal development was normal and the placenta was mostly positioned on the posterior wall in the uterus with sonography. Abdominal and transvaginal ultrasonography demonstrated severe left hydronephrosis and a 17.5 mm hypo-echoic coin lesion surrounded by a hetero-echoic 6 cm mass in the parauterine space (Fig. 1a). Color Doppler ultrasonography revealed a spinning turbulent flow pattern inside the mass lesion (Fig. 1b). Contrast-enhanced computed tomography revealed left uterine artery feeding blood flow to the mass and ureteral obstruction by the mass (Fig. 2a). The hydronephrosis of the left ureter was so severe that the calyceal diameter was over 31 mm (Fig. 2b). These results indicated left hydronephrosis secondary to UAP.

**Fig. 3:**
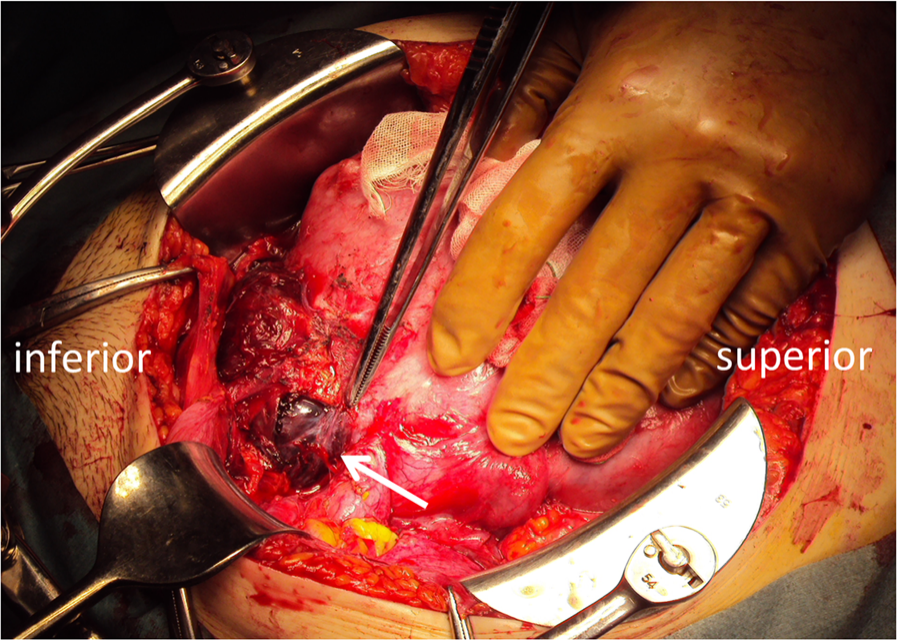
Hematoma formed at the lower left side of the pregnant uterus. The hematoma (approximately 6 cm in size, white arrow) was formed in front of the left-anterior wall of the uterus

The patient underwent laparotomy. The sigmoid colon was firmly adhering to the surface of the uterus and the left pelvic wall. The UAP was buried among the adhesion (Fig. 3). First, ligation of the left internal iliac artery was conducted to block the blood flow to the UAP. Next, exfoliation of the adhesion, removal of the hematoma and detaching of the left ureter from the UAP was carefully performed. Blood loss was controlled because blood flow to the UAP had been blocked. The total blood loss was 870 mL, and the surgical duration was 169 min. The ureteral obstruction was resolved, and the left lower abdominal pain disappeared following surgery. The postoperative course was uneventful, and the patient was discharged 11 days after the operation. She subsequently attended the remaining prenatal check-up series, and the pregnancy followed an uneventful course until early in week 35 of gestation. Fetal growth and Doppler studies were normal. However, from the end of week 35 of gestation, she developed pregnancy-induced hypertension without proteinuria; thus, a cesarean section was performed at 36 + 0 weeks of gestation. A healthy girl of 2257 g was delivered. The Apgar’s scores were 8 and 9 at 1 min and 5 min after birth, respectively. The placenta was mostly positioned on the posterior wall in the uterus. The umbilical artery pH was 7.349. At the cesarean section, the left lower uterine segment where the UAP had previously been present was not visible. She was discharged 7 days after the operation, and post-operative follow-up at 1 month after the delivery was uneventful. Moreover, the infant was also confirmed to have grown well and to be developmentally normal at the one-year examination after birth.

## Discussion

To the best of our knowledge, this is the first report of a symptomatic severe hydronephrosis secondary to UAP diagnosed in the early second trimester of pregnancy. This case highlighted two important clinical issues. First, a UAP formed on the lateral side of the uterus may cause severe symptomatic hydronephrosis from the early second trimester of pregnancy. Secondly, when uterine artery ligation seems impossible, ligation of the internal iliac artery is a serious option for shutting off the blood flow to the UAP in the early second trimester of pregnancy.

We believe that there are two types of UAP. One has communication with the uterine cavity and causes genital bleeding. Another has no communication and forms a hematoma outside of the uterus [9]. The latter type can cause hydronephrosis. During pregnancy, mild dilatation of the renal pelvis and ureter is a common physiologic occurrence, especially on the right side. However, severe symptomatic hydronephrosis of the left kidney in the early second trimester is uncommon [5]. The mean calyceal diameter in the 17th week of gestation is reported as about 5 mm [5], with reported percentages of only 3.9% having maximum calyceal diameters over 15 mm in the left kidney during the second trimester [10]. Our patient’s left hydronephrosis was so severe that the calyceal diameter was over 31 mm (Fig. 3b). Therefore, the hydronephrosis was not thought to be caused only by the gravid uterus. We assumed that the enlarged UAP, gravid uterus, and adhesions, all contributed to the development of hydronephrosis in this case. When we find severe hydronephrosis in early term pregnancies, we should consider causes other than a gravid uterus. In a pregnancy after previous surgical procedures to the uterus, UAP should be considered as one of the possible causes of severe hydronephrosis.

When uterine artery ligation seems to be impossible, ligation of the internal iliac artery is an adequate method to shut off the blood flow to the UAP in the early second trimester of pregnancy. Uterine artery embolization is reported to be effective for UAP during pregnancy [6, 7]. Laubach et al. reported uterine artery embolization for a patient of UAP at 29 weeks of gestational age, and Cornette et al. also reported a similar case. However, neither of their patients demonstrated any pain or hydronephrosis. Furthermore, one of the patients developed placental abruption 5 days after embolization. Our patient showed severe hydronephrosis and strong pain. It was considered that these symptoms would not be improved only by uterine artery embolization. We, therefore, chose surgical intervention. During the surgery, we first had to block the blood flow to the UAP. Selective uterine artery ligation is an efficient method for obstructing the flow to UAP. However, in our case, as the UAP expanded to the left side of the pelvis in addition to the firm adhesion around it, severe hemorrhage might have occurred before the uterine artery was displayed; isolation of the uterine artery was therefore difficult. Ligation of the internal iliac artery is an effective procedure for severe obstetric hemorrhage [11, 12]. The advantages of ligation of the internal iliac artery are that it is easy to perform regardless of the enlarged uterus and it infallibly blocks the flow of the uterine artery. In fact, when we removed the hematoma in the UAP to release the compression of the ureter, serious bleeding was absent. There was no influence on the growth of the fetus in the subsequent pregnancy. We, therefore, believe that ligation of the internal iliac artery is a safe method for both mother and fetus as a treatment for UAP in the early second trimester of pregnancy.

## Conclusions

In conclusion, UAP can cause hydronephrosis in the early second trimester of pregnancy and ligation of the internal iliac artery is an effective procedure for surgical treatment of this condition, when uterine artery ligation seems not possible. When we find severe hydronephrosis in early- to mid-term pregnancies, we should consider causes other than the effect of the pregnancy itself. In pregnancy following previous surgical procedures to the uterus, UAP should be considered in the differential diagnosis of symptomatic hydronephrosis. Myomectomy in women planning to become pregnant should be performed only in severe cases, like possibly this case.

## Abbreviations

UAP: Uterine artery pseudoaneurysm
